# Epigenomic profiling of archived FFPE tissues by enhanced PAT-ChIP (EPAT-ChIP) technology

**DOI:** 10.1186/s13148-018-0576-y

**Published:** 2018-11-16

**Authors:** Stefano Amatori, Giuseppe Persico, Claudio Paolicelli, Roman Hillje, Nora Sahnane, Francesco Corini, Daniela Furlan, Lucilla Luzi, Saverio Minucci, Marco Giorgio, Pier Giuseppe Pelicci, Mirco Fanelli

**Affiliations:** 1 Department of Biomolecular Sciences, Molecular Pathology Laboratory “PaoLa”, University of Urbino “Carlo Bo”, Via Arco d’Augusto 2, 61032 Fano, PU Italy; 2 Department of Experimental Oncology, European Institute of Oncology, Via Adamello 16, 20139 Milan, Italy; 3 Unit of Pathology, Department of Medicine and Surgery, University of Insubria, Via O. Rossi 9, 21100 Varese, Italy; 4 U.O.C. Anatomia Patologica, “C. G. Mazzoni” Hospital, Via degli Iris 2, 63100 Ascoli Piceno, Italy

**Keywords:** Chromatin immunoprecipitation, ChIP-Seq, PAT-ChIP, Pathology samples, FFPE tissues, Formalin fixation

## Abstract

**Background:**

The introduction of pathology tissue-chromatin immunoprecipitation (PAT-ChIP), a technique allowing chromatin immunoprecipitation (ChIP) from formalin-fixed paraffin-embedded (FFPE) tissues, has extended the application of chromatin studies to clinical patient samples. However, extensive crosslinking introduced during routine tissue fixation of clinical specimens may hamper the application of PAT-ChIP to genome-wide studies (PAT-ChIP-Seq) from archived tissue samples. The reduced efficiency in chromatin extraction from over-fixed formalin archival samples is the main hurdle to overcome, especially when low abundant epigenetic marks (e.g., H3K4me3) are investigated.

**Results:**

We evaluated different modifications of the original PAT-ChIP protocol to improve chromatin isolation from FFPE tissues. With this aim, we first made extensive usage of a normal human colon specimen fixed at controlled conditions (24 h, 48 h, and 72 h) to mimic the variability of tissue fixation that is most frequently found in archived samples. Different conditions of chromatin extraction were tested applying either diverse sonication protocols or heat-mediated limited reversal of crosslinking (LRC). We found that, if compared with canonical PAT-ChIP protocol, LRC strongly increases chromatin extraction efficiency, especially when 72-h fixed FFPE samples are used. The new procedure, that we named enhanced PAT-ChIP (EPAT-ChIP), was then applied at genome-wide level using an archival sample of invasive breast carcinoma to investigate H3K4me3, a lowly abundant histone modification, and H3K27me3 and H3K27ac, two additional well-known histone marks.

**Conclusions:**

EPAT-ChIP procedure improves the efficiency of chromatin isolation from FFPE samples allowing the study of long time-fixed specimens (72 h), as well as the investigation of low distributed epigenetic marks (e.g., H3K4me3) and the analysis of multiple histone marks from low amounts of starting material. We believe that EPAT-ChIP will facilitate the application of chromatin studies to archived pathology samples, thus contributing to extend the current understanding of cancer epigenomes and enabling the identification of clinically useful tumor biomarkers.

**Electronic supplementary material:**

The online version of this article (10.1186/s13148-018-0576-y) contains supplementary material, which is available to authorized users.

## Background

In these years, immense developments are occurring in the fields of early cancer detection, biomarker-based treatment selection, and disease response to treatments. A growing contribution to these progresses is coming from the discovery of epigenetic biomarkers (epimarkers) [1–5]. Although translational outcomes are still on the horizon, epimarkers are thought to be the future perspective not only to understand the molecular basis of carcinogenesis but also in cancer diagnosis and potential-targeted treatment.

Chromatin immunoprecipitation (ChIP) is considered one of the most powerful experimental approach to investigate the epigenetic landscape in many biological models. In fact, ChIP allows the study of histone post-translational modifications (HPTMs) which are thought to play a crucial role in epigenetic regulation of gene expression and to contribute, when altered, to cancer development [6–8]. The combination of ChIP with next-generation sequencing (NGS) has permitted the mapping of HPTMs over the entire genome [9–11]. We recently introduced a new ChIP technique, named pathology tissue-chromatin immunoprecipitation (PAT-ChIP) that enables chromatin extraction and immunoprecipitation from formalin-fixed paraffin-embedded (FFPE) tissues, thus allowing the exploitation of a vast number of clinically annotated tissue resources stored in pathology archives [12, 13]. PAT-ChIP can be coupled with the analysis of the epigenetic state of single loci by quantitative PCR (qPCR), or with genome-wide scale studies by NGS techniques (PAT-ChIP-Seq). In addition, we demonstrated that PAT-ChIP can be coupled with laser capture microdissection (LCM) to study more homogeneous cellular populations [14]. Since its introduction, the technique has been applied by several investigators [15–23] giving new impetus to chromatin studies in patient samples and to the identification of new potential epimarkers in function of the clinical information of patients.

However, we have experienced that genome-wide studies from FFPE archival samples can be hindered by the low efficiency of chromatin isolation, often due to extensive tissue fixation introduced during routine pathological processing. A solution containing 3.7–4% of formaldehyde (FA) is routinely used as fixative reagent with a length of fixation that is influenced by different factors (e.g., day of tissue resection, operators/instrument availability, etc.) [24]. Despite the recent advancements in the standardization of FFPE tissue preparation, the times of fixation are extremely variable, normally ranging from 24 to 72 h [25–27].

FA is a tight (2 Å) crosslinking agent that efficiently produces both protein–nucleic acid and protein–protein crosslinks. Amino and imino groups of amino acids (lysines, arginines, and histidines) and of DNA (primarily adenines and cytosines) readily react with FA forming a Schiff’s base that can participate in a second linkage with an additional amino group and condense to give the final DNA–protein complex [28–30]. Extensive crosslinking to which FFPE archival samples can be exposed produces a dense network of crosslinked cellular biomolecules that can render chromatin extraction extremely challenging.

In this work, we investigated the possibility to improve chromatin extraction efficiency from FFPE tissues to facilitate the combination of the PAT-ChIP protocol with NGS technology, allowing genome-wide studies using clinical archival samples.

## Materials and methods

### Preparation of FFPE tissues

Specimens of normal colon tissue were obtained from a patient affected by colorectal cancer who underwent curative surgical resection at the Ospedale di Circolo, ASST Sette Laghi (Varese, Italy). After collection (10 cm distant from the tumor), the tissue was divided in three pieces of similar size and fixed for 24 h, 48 h, or 72 h. All tissues were fixed in neutral-buffered formalin (formaldehyde 4% wt/vol and acetate buffer 0.05 M) and routinely processed in paraffin wax using the automated tissue processor Donatello (Diapath, Bergamo, Italy).

### Chromatin extraction from FFPE tissues by standard PAT-ChIP

Chromatin extraction at standard conditions was performed following the already described PAT-ChIP procedure [12–14]. Briefly, four FFPE tissue sections of 10 μm thickness were first deparaffinized by histolemon solution (Carlo Erba, Milan, Italy) and dehydrated by decreasing concentrations of ethanol. Sections were then lysed, fragmented by mild sonication, and subjected to controlled micrococcal nuclease (MNase) digestion. Chromatin extraction was performed using a canonical sonicator (EpiShear from Active Motif, Carlsbad, CA, USA) with pulses of sonication of 5 s interrupted by pauses of 10 s. Sonication profiles were as follows: Std (18 pulses of 5 s at 85% of amplitude), 75 × 54 (54 pulses of 5 s at 75% of amplitude), and 65 × 54 (54 pulses of 5 s at 65% of amplitude).

### Chromatin extraction from FFPE tissues by EPAT-ChIP

In the enhanced PAT-ChIP (EPAT-ChIP) procedure, digestion of chromatin with MNase was removed and a limited reversal of crosslinking (LRC) step was added prior of chromatin extraction. Sonication conditions were also adapted in consequence of the partial de-crosslinking of the samples.

In detail, FFPE tissue sections of 10 μm thickness were first deparaffinized by five sequential incubations, of 10 min each, in 1 ml of histolemon solution (Carlo Erba, Milan, Italy) at room temperature. When not specified, all centrifugations were performed at 17,860×*g* for 3 min at + 4 °C. Samples were rehydrated by decreasing concentrations of ethanol starting from 100% through to 95%, 70%, 50%, 20% and water (10 min at room temperature for each step in 1 ml). Samples were then resuspended in 0.5 ml of lysis buffer (10 mM Tris-HCl pH 7.4, 0.15 M NaCl, 3 mM CaCl_2_, 2 mM MgCl_2_, 0.5% Tween20, 1 mM PMSF, and 10 μg/mL RNase A—Roche, Mannheim, Germany) and incubated 30 min at room temperature on a rotating platform. After resuspension in 0.3 ml of fragmentation buffer (50 mM Tris-HCl, pH 7.4, 0.32 M sucrose, 4 mM MgCl_2_, 1 mM CaCl_2_, and 0.1 mM PMSF), sections were fragmented by sonicating three times for 30 s (60 s off), in a thermoblock refrigerated at − 20 °C, with an amplitude of 40% using the EpiShear sonicator (Active Motif, Carlsbad, CA, USA). All sonications were performed using a 3.2 mm probe. LRC was conducted by resuspending the sample in 1 ml of sodium citrate buffer (10 mM sodium citrate, 0.05% Tween20, pH 6.0) followed by an incubation of 1 h at + 80 °C.

For chromatin isolation, samples were resuspended in 0.4 ml of extraction buffer (10 mM Tris-HCl pH 7.4, 0.15 M NaCl, 3 mM CaCl_2_, 2 mM MgCl_2_, 0.1% SDS) and sonicated at 40% of amplitude with three pulses of 30 s each, interrupted by 60 s pauses, in a thermoblock refrigerated at − 20 °C. After being cleared by centrifugation (9500× *g* for 5 min at room temperature), supernatants containing chromatin were saved and an aliquot of 40 μl (corresponding to the 10% of total isolated chromatin or “input”) was subjected to a complete de-crosslinking through overnight (16 h) incubation at + 65 °C in the presence of 0.2 M NaCl, followed by digestion with 0.1 mg/ml proteinase K (3 h at + 45 °C). DNA purification was carried out using the PCR Purification Kit (Qiagen, Hilden, Germany) following manufacturer’s instructions and DNA was fluorimetrically quantified by Qubit (Invitrogen, Eugene, OR, USA) using the dsDNA HS Assay Kit (Invitrogen, Eugene, OR, USA) to estimate the total quantity of chromatin present in supernatants (input).

Chromatin fragmentation was also checked by electrophoretic separation, on a 1.3% agarose gel, of at least 50 ng of purified input DNA as previously described [31]. DNA was stained with SYBR Gold stain (Invitrogen, Eugene, OR, USA).

### Chromatin immunoprecipitation and DNA isolation

Chromatin was immunoselected in incubation buffer (30 mM Tris-HCl pH 7.4, 50 mM NaCl, 5 mM Na_2_EDTA, and 0.1 mM PMSF), in a final volume of 0.5 ml, for 16 h at + 4 °C on a rotating platform using the following antibodies: anti-H3K4me3 (2 μl of whole serum extract—39159, Lot. 01609004; Active Motif, Carlsbad, CA, USA), anti-H3K27ac (1.25 μg of immunogen affinity-purified antibody—ab4729, Lot. GR254707-1; Abcam, Cambridge, UK), or anti-H3K27me3 (4 μg of protein A-purified antibody—07-449, Lot. JBC1873477; Millipore, Temecula, CA, USA) antibodies. Then, 40 μl of 50% (vol/vol) slurry rec-Protein G-Sepharose 4B Conjugate (preincubated 16 h at + 4 °C with 1 mg/ml of BSA in incubation buffer; Invitrogen, Frederick, MD, USA) were added to each ChIP assay and incubated for 3 h at + 4 °C. After centrifugation (1270×*g* for 2 min at + 4 °C), pellets were sequentially washed with 10 ml of cold washing buffer A (50 mM Tris-HCl pH 7.4, 1% TritonX-100, 50 mM NaCl, 5 mM Na_2_EDTA, and 0.1 mM PMSF), 10 ml of cold washing buffer B (50 mM Tris-HCl pH 7.4, 1% TritonX-100, 100 mM NaCl, 5 mM Na_2_EDTA, and 0.1 mM PMSF), and 10 ml of cold washing buffer C (50 mM Tris-HCl pH 7.4, 1% TritonX-100, 150 mM NaCl, 5 mM Na_2_EDTA, and 0.1 mM PMSF). Each wash was performed by inverting the tubes 25 times. Elution was carried out by adding 0.3 ml of elution buffer (Tris-EDTA buffer, 1% SDS) and incubating for 30 min at room temperature in a rotating platform. After centrifugation (1270×*g* for 2 min at + 4 °C), the supernatant was saved and the elution repeated with only 50 μl of elution buffer (by vortexing 10 s at maximum speed) to obtain a final volume of 0.35 ml (the “bound” fraction).

Bound fractions, and an amount corresponding to 5% of previously saved inputs, were de-crosslinked, purified, and quantified (triplicate readings) as described above.

Student’s *t* test was used for comparing differences between two groups, and one-way ANOVA followed by Tukey’s HSD test for comparing differences between multiple groups.

### Locus-specific analysis of immunoselected (bound) DNA

Purified DNA from bound and 5% input fractions was analyzed in triplicate by real-time quantitative PCR (qPCR) using the Fast Start SYBR Green Master Mix (Roche, Mannheim, Germany) and the Rotor-Gene 6000 robocycler (Corbett Life Science, Sydney, Australia) as already reported [32]. Amplifications were carried out using conditions already described [14] and primer pairs reported in Table 1. Data are presented as percentage of enrichment with respect to the input.

**Table 1:** Sequences of primers employed for real-time qPCR assay

Gene	Forward primer sequence	Forward primer sequence	Start	End
			Respect TSS (bp)	
Vcl	5′-ATGCCAGTGTTTCATACGCG-3′	5′-CGCCCTCCTCGTGCATTAT-3′	+ 94	+ 184
Gapdh	5′-TTCGCTCTCTGCTCCTCCTG-3′	5′-CCTAGCCTCCCGGGTTTCTC-3′	+ 95	+ 185
Hapln1	5′-TCGGATGCTCTCAAGTTCTGC-3′	5′-TCGCCCAGAGACAAACTTAAGG-3′	+ 177	+ 267
Col2a1	5′-CTTTCGAGGCTGGCGAACT-3′	5′-CGGTTCAGGTTACAGCCCA -3′	− 86	− 16

### Pipeline of ChIP-Seq analysis

Input ChIP DNA was blunt-ended and phosphorylated, and a single “A” nucleotide was added to the 3′ ends of the fragments in preparation for ligation to adapters that have a single-base “T” overhang using enzymes and reagents from NEB (New England Biolabs, Ipswich, MA, USA). The ligation products were purified and size-selected by Agencourt AMPure XP beads (Beckman Coulter, Beverly, MA, USA). Purified DNA was PCR-amplified with PfuUltra II Fusion HS DNA Polymerase (Agilent Technologies, Santa Clara, CA, USA) to enrich for fragments that have adapters on both ends. All these steps were performed on the automation instrument Biomek FX (Beckman Coulter, Beverly, MA, USA). The final purified product was then quantitatively and qualitatively checked on Bioanalyzer 2100 (Agilent Technologies, Santa Clara, CA, USA). Libraries with distinct adapter indexes were multiplexed (1/5 libraries per lane) and after cluster generation on FlowCell were sequenced for 50 bases in the single read mode on a HiSeq 2000 sequencer (Illumina Inc., San Diego, CA, USA).

Reads were aligned to hg19 using bowtie (version 0.6.2-r126). Unmapped reads, reads with a MAPQ smaller than 1, and duplicate reads were removed using samtools (version 0.1.18). Mapped sequence reads were extended to 200 bp, which was the estimated mean insert size targeted in the size selection step when preparing the libraries, using deepTools (version 2.5.4).

Detection of peaks was performed using MACS2 software from Galaxy browser (https://usegalaxy.org—bdgpeakcall function to call narrow peaks from H3K4me3 and H3K27ac tracks and MACS2 bdgbroadcall function for broad peaks from H3K27me3). Intersections between genomic regions were performed using the specific function on Galaxy browser. The R/Bioconductor package ChIPseeker [33] was used to annotate the genomic features of peaks, while data sets and peaks were visualized on UCSC Genome Browser (https://genome.ucsc.edu) from where snapshots were taken.

### Immunofluorescence

HeLa cells were harvested and fixed with 1% formaldehyde in PBS for 10 min at + 37 °C. Immunofluorescence was performed following the same conditions used in the PAT-ChIP assay (buffers, incubation timing, and temperature) as previously described [13]. Briefly, after permeabilization with lysis buffer, cells were subjected to LRC by heating 1 h at + 80 °C in sodium citrate buffer and spotted by cytospin. Cells were then blocked with FBS and incubated in a humidified chamber with the same antibodies and concentrations used in ChIP experiments (anti-H3K4me3, anti-H3K27ac, and anti-H3K27me3). After washing with ice-cold washing buffer A, washing buffer B, and washing buffer C, cells were incubated with a fluorochrome-conjugated secondary antibody (Donkey anti-rabbit IgG highly cross-adsorbed secondary antibody, Alexa Fluor 488—ThermoFisher Scientific, San Jose, CA, USA), washed with PBS, and counterstained with DAPI as previously described [13].

Fluorescence signal was acquired using an Olympus BX51 microscope equipped with an Olympus F-View II digital camera and AnalySIS software (Soft Imaging System, GmbH).

## Results

### Limited reversal of crosslinking increases the amount of soluble chromatin isolated from FFPE samples at different times of fixation

Although attempts to standardize the times of fixation to 24 h/48 h for most tissue types, samples derived from surgery are in most cases still crosslinked with 3.7–4% FA for times usually ranging between 24 and 72 h [25–27]. We prepared tissues at different times of fixation to mimic the conditions of formalin fixation routinely found in archival samples. We chose to use normal human colon tissue as a model due to the availability of high quantities of this human material as scrap from colorectal surgeries. The tissue was divided in three different pieces of comparable size, crosslinked for 24 h, 48 h, or 72 h and included in paraffin. Four sections of 10 μm thickness, and about 1 cm^2^ of area (equivalent to a total of 4 mm^3^) were used for each extraction condition and subsequent chromatin immunoprecipitation.

**Fig. 1:**
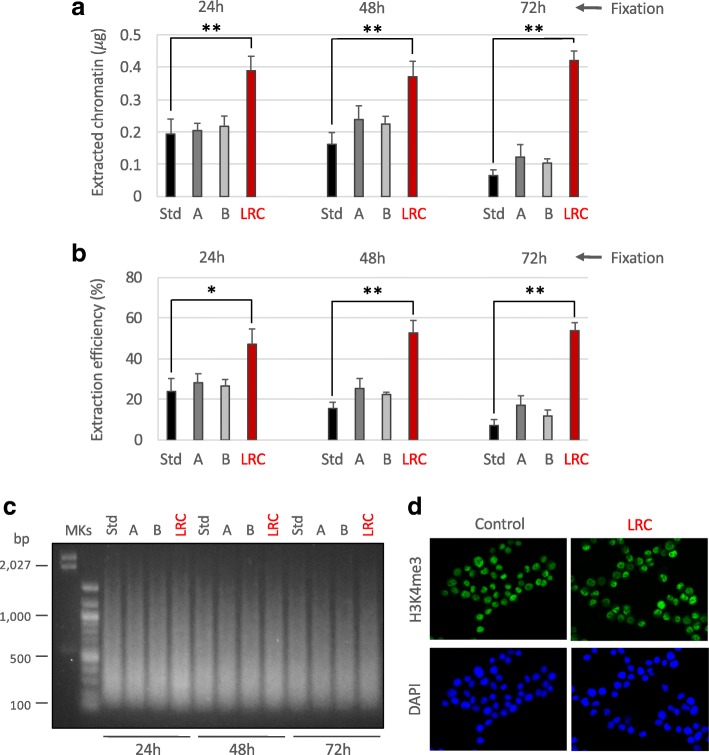
Attempts to improve chromatin extraction from FFPE samples at different times of fixation. Different conditions of chromatin extraction from normal colon FFPE tissues fixed at the times reported in figure were tested. The total amount of isolated chromatin was fluorimetrically evaluated after chromatin de-crosslinking and DNA purification (**a**) while the extraction efficiency was calculated considering the amount of DNA of extracted chromatin compared to the total DNA present in the sample (**b**). Std: standard PAT-ChIP, 18 pulses of sonication of 5 s at 85% of amplitude; A: 54 pulses of sonication of 5 s at 75% of amplitude; B: 54 pulses of sonication of 5 s at 65% of amplitude; LRC: condition in which the sample was subjected to limited reversal of crosslinking, 3 pulses of sonication of 30 s at 40% of amplitude. *P < 0.05 with respect to standard condition for each time of fixation by one-way ANOVA with Tukey’s HSD. **P < 0.01 with respect to standard condition for each time of fixation by one-way ANOVA with Tukey’s HSD. All the experiments were conducted in triplicates. **c** Evaluation of chromatin fragmentation by electrophoretic separation on 1.3% agarose gel electrophoresis (AGE) followed by SYBR Gold staining of purified input DNA. MKs, molecular weight markers. **d** Compatibility of LRC with H3K4me3 immunoselection. HeLa cells were subjected to formaldehyde fixation and treated with LRC or left untreated. Cells were stained by immunofluorescence with anti-H3K4me3 antibody (green, upper panels) following the same procedure described for the PAT-ChIP assay (buffers, timing, and temperature of incubations) and with DAPI to label nuclei (blue, lower panels)

We already observed in preliminary experiments that the efficiency of chromatin extraction from archival samples, using the standard PAT-ChIP procedure, is low and may decrease in function of prolonged tissue fixation (data not shown) hindering the application of genome-wide studies (PAT-ChIP-Seq). Since tissue fixation of archival FFPE samples cannot be controlled by definition, we focused on the improvement of chromatin extraction efficiency by (i) modifying sonication steps in terms of times and amplitudes relative to the standard (Std) procedure and (ii) performing a heat-mediated limited reversal of crosslinking (LRC) of the tissue with the intent to reduce the chromatin complexity and facilitating its subsequent isolation by sonication. As expected, we found that chromatin extraction efficiency decreases with the increase of the time of fixation: less than 200 ng (equal to 23.9% of total DNA) isolated from the 24-h fixed sample and only 65 ng (equal to 7.2% of total DNA) from the 72-h fixed sample (Fig. 1a, b).

We previously found that prolonged sonication at high amplitudes (85% or more) significantly decreases the efficiency of immunoselection, reasonably due to epitope damaging (data not shown). Thus, we evaluated the possibility of extending the timing of extraction proposed in the Std protocol (from 18 pulses of 5 s each—a total of 1 min and 30 s to 54 pulses of 5 s each—a total of 4 min and 30 s) by decreasing the amplitude of sonication (from 85 to 75 and 65%). However, we observed just a little, not significant, increase of chromatin extraction efficiency (Fig. 1a, b). Chromatin extracted at these new conditions showed levels of fragmentation comparable to the one extracted using the Std conditions of sonication (Fig. 1c).

Considering that the main hurdle in extracting chromatin from FFPE tissues is represented by extensive formalin fixation, we evaluated the possibility of improving extraction by reducing the structural complexity generated by FA fixation through heat-mediated LRC. Different LRC conditions were tested, varying in temperature (from + 65 to + 95 °C), time of incubation (from 10 min to 16 h), as well as buffer pH (from 6.0 to 9.0). At the end of this testing period, the condition of +80 °C for 1 h of incubation in sodium citrate buffer pH 6.0 was selected as the most potent to improve chromatin extraction, without interfering with the recognition of the epitope (data not shown). We found that LRC significantly increased the efficiency of chromatin extraction from the human colon specimens in all the conditions of FA fixation (24 h, 48 h, and 72 h). Notably, the best increment of chromatin isolation efficiency was monitored in the sample fixed for 72 h, which reached the same amount of chromatin obtained from samples fixed for lower times (24 h/48 h of fixation—Fig. 1a, b—red columns). In consequence of the LRC-mediated reduction of chromatin complexity, the digestion with MNase (proposed in the original PAT-ChIP protocol) was eliminated and the sonication conditions for chromatin extraction were changed compared to the standard procedure (by decreasing the amplitude of sonication from 85% to 40%) to avoid excessive chromatin fragmentation (Fig. 1c).

To preliminarily test if LRC could interfere with the recognition of the epitope by the antibody, we used the epigenetic mark H3K4me3. The impact of LRC on epitope recognition was first evaluated using an immunofluorescence approach conducted in the same conditions used in the PAT-ChIP protocol (buffers, temperatures, times of incubation). Fixed HeLa cells were subjected to LRC for 1 h at + 80 °C in sodium citrate buffer pH 6.0 and stained with the anti-H3K4me3 antibody. Subsequent results show a signal that is quantitatively and qualitatively comparable to that of the control (not LRC-treated) sample (Fig. 1d).

### Chromatin extracted by limited reversal of crosslinking is suitable for immunoprecipitation and assures higher amounts of final DNA

**Fig. 2:**
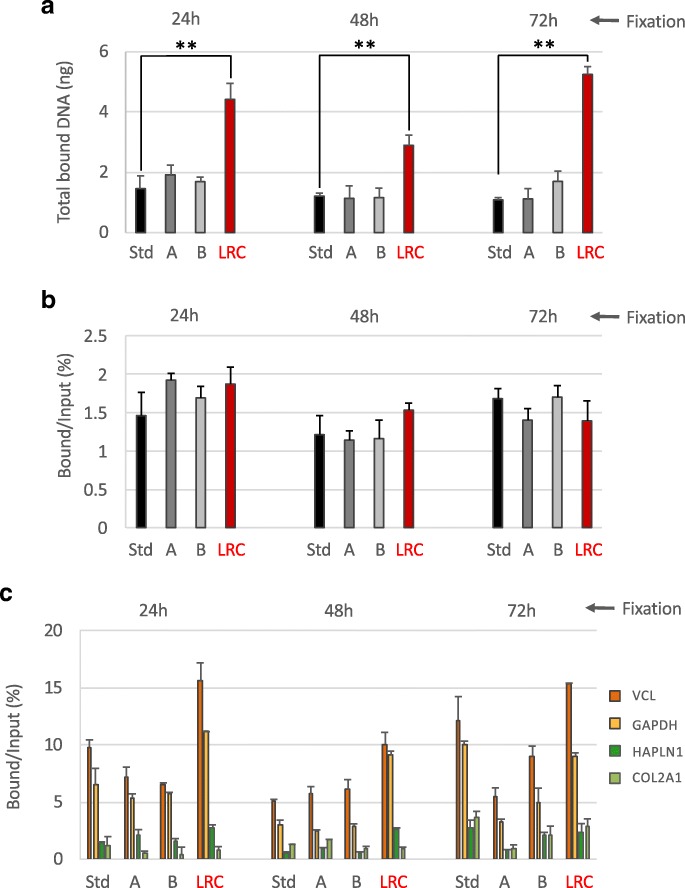
Immunoselection compatibility of chromatin isolated using different extraction strategies. Chromatin from normal colon FFPE samples at different times of fixation, extracted following the different strategies described above, was immunoprecipitated with an anti-H3K4me3 antibody. After immunoselection, chromatin was de-crosslinked and the DNA purified and fluorimetrically quantified (**a**). Input fractions were also purified and the percentage of enrichment by the antibody compared to the input was calculated (**b**). Transcriptionally active (VCL and GAPDH) and inactive (HAPLN1 and COL2A1) promoter regions were amplified by real-time qPCR to evaluate the specificity of the immunoselection. H3K4me3 enrichments are expressed as percentage of bound compared to the input (**c**). Mock (no antibody) control did not produce amplification. Std: standard PAT-ChIP, 18 pulses of sonication of 5 s at 85% of amplitude; A: 54 pulses of sonication of 5 s at 75% of amplitude; B: 54 pulses of sonication of 5 s at 65% of amplitude; LRC: condition in which the sample was subjected to limited reversal of crosslinking, 3 pulses of sonication of 30 s at 40% of amplitude. **P < 0.01 with respect to standard condition for each time of fixation by one-way ANOVA with Tukey’s HSD. All the experiments were conducted in triplicates

Once extracted using the different conditions described above, chromatin was checked for immunoselection by the anti-H3K4me3 antibody. This HPTM was chosen for different reasons: (i) first of all, it represents a good “stress-test” for the procedure since immunoselection against H3K4me3 normally produces poor amounts of final DNA, in particular when low quantities of input chromatin are used, increasing the chances of failure in the generation of NGS libraries; (ii) secondly, its close association with gene promoters and its narrow distribution allows a better measure of ChIP-Seq specificity and resolution. As shown in panels a and b of Fig. 2, we found that chromatin extracted using the different conditions showed comparable efficiency of immunoselection (with percentages of enrichment compared to the input ranging between 1.12 and 1.92%). However, the quantity of final DNA was significantly greater in samples treated with limited reversal of crosslinking (LRC) in consequence of the usage of higher amounts of input chromatin (isolated from only four FFPE sections of 10 μm, see “Materials and Methods” section for details).

The specificity of the immunoselection was preliminarily analyzed by real-time qPCR, measuring the enrichment of the promoter regions of two genes known to be ubiquitously expressed (vinculin—VCL and glyceraldehyde-3-phosphate dehydrogenase—GAPDH) and two genes known to be inactive (hyaluronan and proteoglycan link protein 1—HAPLN1 and collagen, type II, alpha 1—COL2A1). As shown in panel c of Fig. 2, we found that the enrichment of active over silent gene promoters is observable at all the conditions tested.

### The new LRC-based technique (EPAT-ChIP) can be used to investigate real archival FFPE samples

**Fig. 3:**
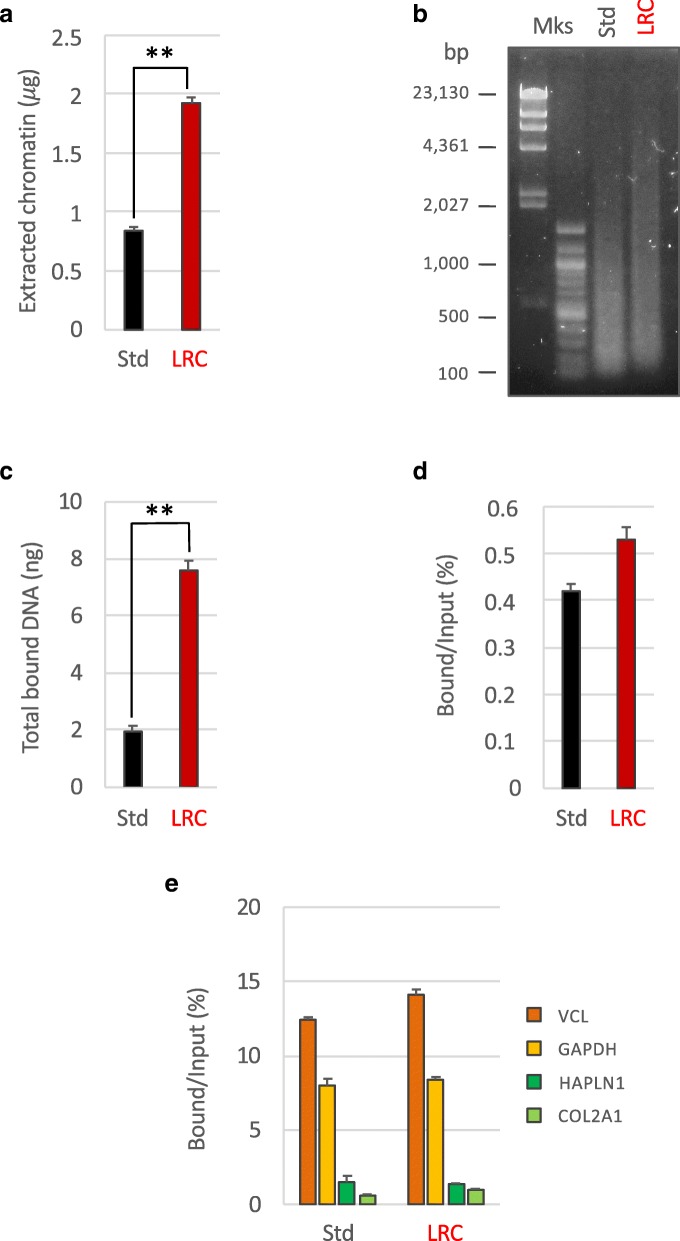
Application and validation of EPAT-ChIP. Chromatin was extracted from an archival invasive breast carcinoma sample by both the standard PAT-ChIP procedure (Std) and the new LRC-based procedure (LRC). The amount of extracted chromatin was estimated by fluorimetric quantitation of purified DNA after complete de-crosslinking (**a**) and chromatin fragmentation was evaluated by electrophoretic separation on 1.3% AGE followed by SYBR Gold staining of purified input DNA (**b**). Chromatin was then subjected to immunoselection with an anti-H3K4me3 antibody, de-crosslinked, and the DNA purified and quantified (**c**). Input fractions were also purified and the percentage of enrichment with respect to the input was calculated (**d**). Transcriptionally active (VCL and GAPDH) and inactive (HAPLN1 and COL2A1) promoter regions were amplified by real-time qPCR (each sample amplified in triplicate) to evaluate the specificity of the immunoselection. H3K4me3 enrichments are expressed as percentage of bound respect to the input (**e**). Mock (no antibody) control did not produce amplification. **P < 0.01 with respect to standard condition by Student’s *t* test

In light of the observations described above, the new procedure modified through the introduction of LRC (1 h at + 80 °C in sodium citrate buffer) was named enhanced PAT-ChIP (EPAT-ChIP). EPAT-ChIP was then applied for validation using an archival invasive breast carcinoma (IBC) FFPE sample. Chromatin was extracted starting from four sections of about 4 cm^2^ of tissue area (for a total volume of 16 mm^3^) following both standard PAT-ChIP and EPAT-ChIP protocols. Even in this case, extraction of chromatin from the sample subjected to LRC produced a higher amount of chromatin with respect to the standard procedure (Fig. 3a), and an average size of chromatin fragments compatible with the ChIP assay (Fig. 3b). Chromatin was then subjected to immunoselection using, in a first instance, an anti-H3K4me3 antibody. The immunoprecipitation conducted using chromatin isolated with LRC produced higher amounts of DNA (a mean of 7.6 ng), and showed comparable efficiency of immunoselection, compared to that performed with chromatin isolated with the standard procedure (a mean of 1.95 ng—Fig. 3c, d). The enrichment of both active and silent gene promoters was analyzed using real-time qPCR, as described above, producing similar results (Fig. 3e).

### EPAT-ChIP can be coupled with NGS for the epigenomic profiling of archival samples

**Fig. 4:**
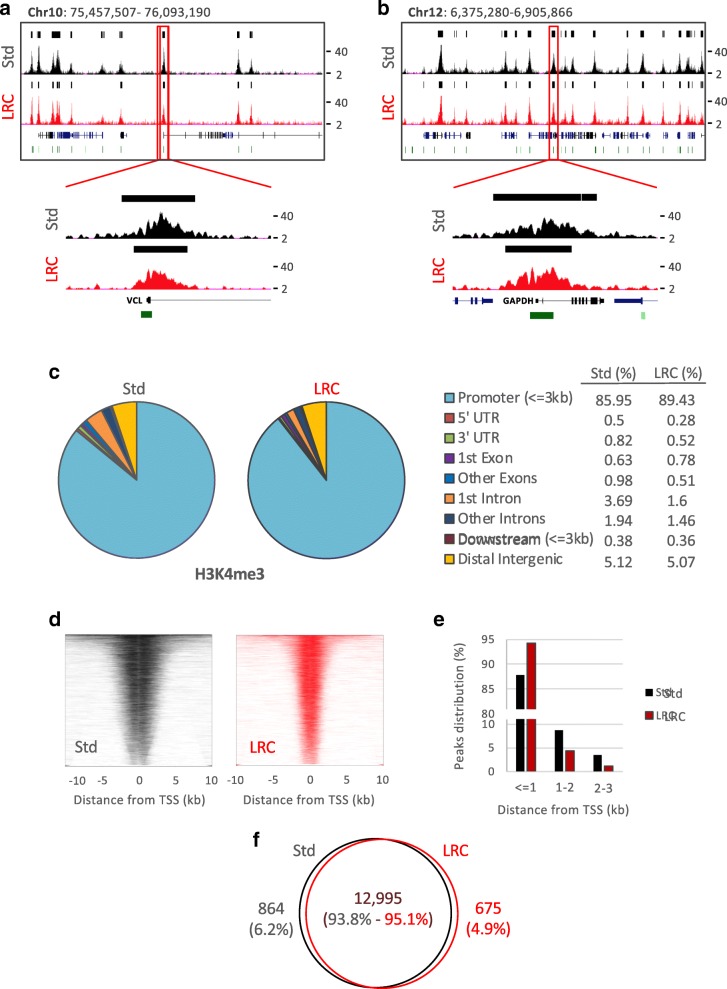
Analysis of genome-wide distribution of H3K4me3 by EPAT-ChIP. Purified DNA previously immunoselected from an archival invasive breast carcinoma sample was subjected to massive parallel sequencing. Snapshots of ChIP-Seq data from UCSC Genome Browser showing the correspondence between standard PAT-ChIP (Std) and EPAT-ChIP (LRC) signals at promoters of the active genes VCL (**a**) and GAPDH (**b**) previously amplified by real-time qPCR. Identified peaks (black bars) are marked above the corresponding profile, CpG islands are reported as green bars, and Ref-Seq genes are indicated in blue. **c** Pie charts depicting the distribution across genomic features with relative percentage values shown on the right. Promoters are defined as − 3 Kb to + 3 Kb relative to the TSS, while Downstream as − 3 Kb relative to the end of 3′ UTR region. **d** Heatmaps illustrating H3K4me3 read densities from − 10 Kb to + 10 Kb relative to the TSS. **e** Distribution of H3K4me3 promoter peaks relative to the TSS. **f** Venn diagram showing common and unique peak-containing promoters identified by standard PAT-ChIP and EPAT-ChIP

We investigated the compatibility of EPAT-ChIP with NGS and compared the new procedure with the Std protocol. Libraries were successfully produced with DNA obtained applying both PAT-ChIP protocols and sequenced. We called 17,041 and 14,041 peaks from the Std PAT-ChIP and EPAT-ChIP data sets, respectively. Peaks from both data sets showed similar distribution of H3K4me3 enrichments (peaks) in correspondence of the promoter of active genes and associated CpG islands (Fig. 4a–d). Peaks from the same regions previously analyzed by qPCR (VCL and GAPDH) are shown in detail to support the correspondence between qPCR and NGS analyses (Figs. 4a, b and 3e—see also snapshots of the two amplified inactive genes in Additional file 1).

Overall, peaks obtained applying both PAT-ChIP protocols are mainly located in correspondence of gene promoters (89.43% and 85.95% for EPAT-ChIP and standard protocols, respectively—Fig 4c). However, while 94.29% of total promoter peaks from EPAT-ChIP are located within 1 Kb from TSS, only 87.77% peaks from canonical PAT-ChIP are located within the same region, suggesting that EPAT-ChIP signal resolution is higher compared to that obtained by the Std PAT-ChIP protocol (Fig. 4d, e).

Finally, we compared the promoters identified as enriched by H3K4me3 immunoselection demonstrating that 93.8% and 95.1% of gene promoters are common in standard protocol and EPAT-ChIP, respectively (Fig. 4f).

To further confirm our results, we applied the same analytical pipeline that we used for our data sets to a H3K4me3 data set from human mammary epithelial cells (HMEC) available from UCSC Genome Browser [33] (GEO accession number: GSM733712). Here, we obtained similar results in terms of peaks overlap with genomic features (Additional file 2a). Interestingly, peaks from the HMEC data set are tightly associated with TSS, showing a distribution comparable to that observed in EPAT-ChIP data set (Additional file 2b).

### Application of EPAT-ChIP to investigate the genome-wide distribution of other histone marks (H3K27me3 and H3K27ac) in archival samples

**Fig. 5:**
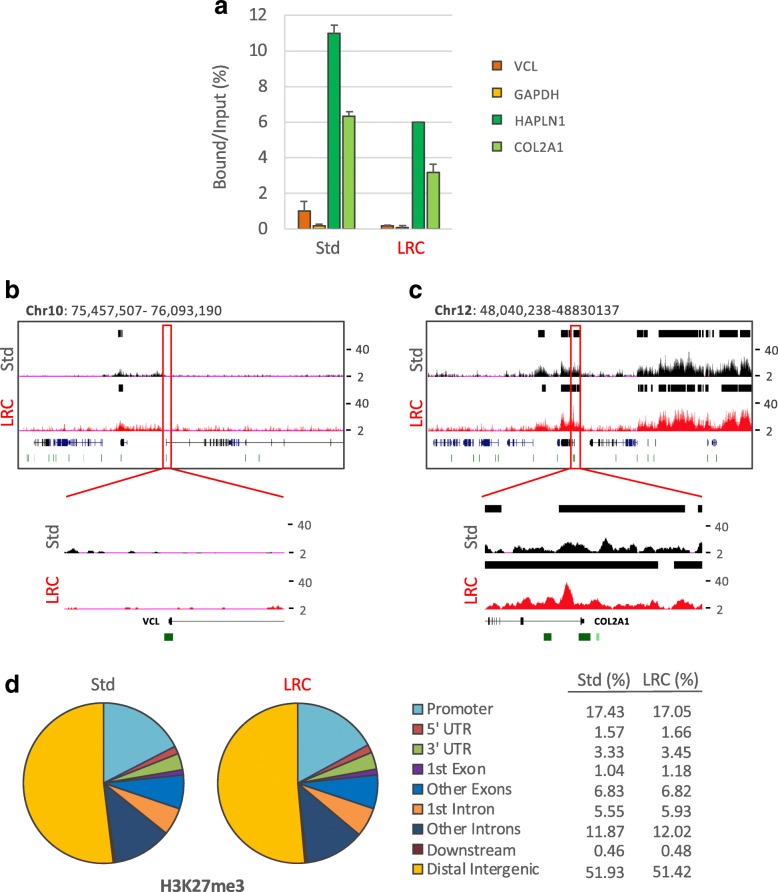
Genome-wide distribution of H3K27me3 by EPAT-ChIP. Genome-wide analysis of the archival invasive breast carcinoma sample was extended to H3K27me3 HPTM. **a** Transcriptionally active (VCL and GAPDH) and inactive (HAPLN1 and COL2A1) promoter regions were amplified by real-time qPCR (each sample amplified in triplicates) to evaluate the specificity of the immunoselections. Enrichment is expressed as percentage of bound DNA with respect to the input. Snapshots showing ChIP-Seq signals at promoters of the active VCL gene (**b**) and the silent COL2A1 gene (**c**) previously amplified by real-time qPCR were taken from UCSC Genome Browser. Identified peaks (black bars) are marked above the corresponding profile, CpG islands are reported as green bars, and Ref-Seq genes are indicated in blue. **d** Pie charts depicting the distribution of peaks across genomic features with relative percentage values shown on the right. Promoters are defined as − 3 Kb to + 3 Kb relative to the TSS, while Downstream as − 3 Kb relative to the end of 3′ UTR region

Finally, we analyzed whether the results obtained by immunoprecipitation of H3K4me3 by EPAT-ChIP can be extended to other histone marks that are functionally different from H3K4me3. We therefore extracted chromatin from the IBC sample (four sections of about 4 cm^2^ of surface for a total volume of 16 mm^3^) using both standard PAT-ChIP and EPAT-ChIP protocols. Chromatin was then subjected to immunoselection against both H3K27me3 and H3K27ac, two well characterized histone marks known to be associated with silent and actively transcribing gene promoters and enhancers, respectively. Also in this case, sufficient amounts of DNA were obtained (Additional file 3) for subsequent library preparation. DNA immunoprecipitated from both chromatin preparations was analyzed by real-time qPCR to preliminarily check the specificity of the immunoselection in the promoter region of two active and two silent genes. Even in this case (H3K27me3), samples processed using the two experimental procedures showed a similar behavior, with silent genes exhibiting high enrichments of this histone mark compared to active genes (Fig. 5a). Libraries were then successfully produced and sequenced, and the distribution of peaks was consistent between the two techniques and with what has been already described in the literature (Fig. 5b–d). H3K27me3 peaks are primarily located in distal intergenic regions (51.93% and 51.42% for Std procedure and EPAT-ChIP, respectively) and in gene bodies/promoters (42.72% and 43% for Std procedure and EPAT-ChIP, respectively—Fig. 5d). Details of snapshots of one expressed gene (VCL) and one silent gene (COL2A1) are shown to further demonstrate the correspondence between real-time qPCR and NGS results (Fig. 5a–c). Similar results were obtained applying the same analytical pipeline that we used for our data sets to a H3K27me3 data set obtained using HMEC available from UCSC Genome Browser [34] (GEO accession number: GSM733722—Additional file 4a).

**Fig. 6:**
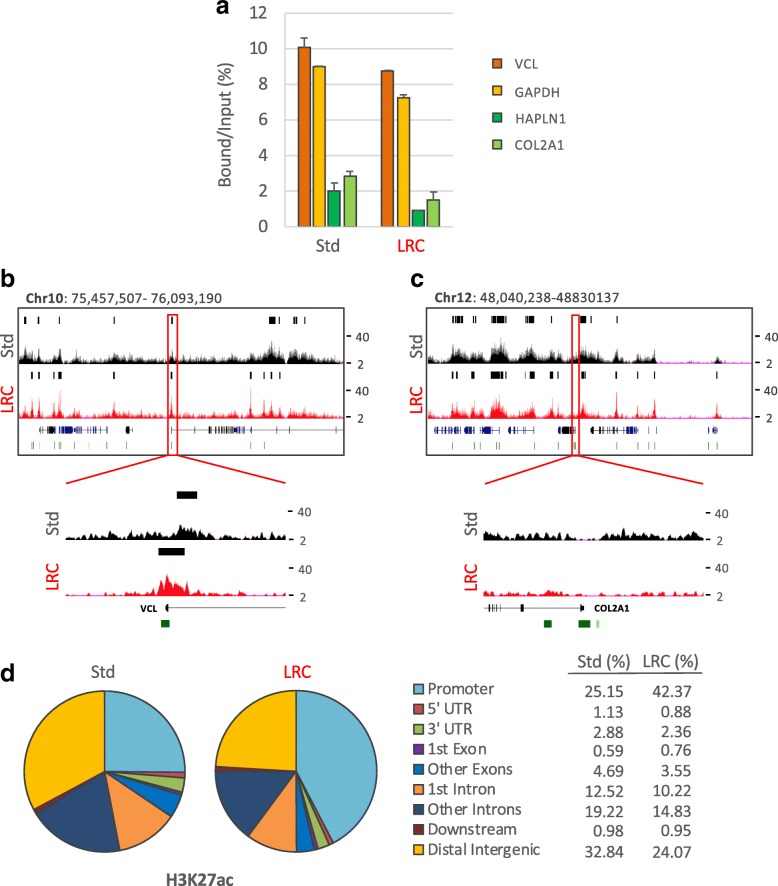
Genome-wide distribution of H3K27ac by EPAT-ChIP. H3K27ac distribution was also investigated genome-wide in the archival IBC sample. **a** Transcriptionally active (VCL and GAPDH) and inactive (HAPLN1 and COL2A1) promoter regions were amplified by real-time qPCR (each sample amplified in triplicates) to evaluate the specificity of the immunoselection. Enrichment is expressed as a percentage of bound DNA with respect to the input. Snapshots showing ChIP-Seq signals at promoters of the active VCL gene (**b**) and the silent COL2A1 gene (**c**) previously amplified by real-time qPCR were taken from UCSC Genome Browser. Identified peaks (black bars) are marked above the corresponding profile, CpG islands are reported as green bars, and Ref-Seq genes are indicated in blue. **d** Pie charts depicting the distribution of peaks across genomic features with relative percentage values shown on the right. Promoters are defined as − 3 Kb to + 3 Kb relative to the TSS, while Downstream as − 3 Kb relative to the end of 3′ UTR region

The qPCR analysis of the H3K27ac immunoselected DNA was performed showing, as expected, the enrichment of active compared to silent genes promoters (Fig. 6a). Peaks of enrichment were identified after sequencing, showing a distribution mainly focused on promoters and intergenic regions (Fig 6b–d). However, the sample processed by Std PAT-ChIP showed an inferior quality of both read profiles (Fig. 6b, c) and peaks distribution, with only 25.15% of peaks located on gene promoters and 32.84% of peaks located on distal intergenic regions (Fig. 6d). Interestingly, EPAT-ChIP processing was able to strongly improve the quality of H3K27ac profile (Fig. 6b, c) producing a distribution of peaks comparable to that obtained from a HMEC H3K27ac data set available from the UCSC Genome Browser [34] (GEO accession number: GSM733660—Additional file 4b).

## Discussion

The introduction of PAT-ChIP technology opened the door to the study of archival FFPE samples, which represent an extraordinary source of epigenomic information to investigate the epigenetic basis of cancer and other diseases, as well as to identify new potential epigenetic biomarkers. Despite numerous attempts at standardization, the processing of FFPE tissues is still extremely variable. In particular, the duration of FA fixation to which tissues are normally subjected prior to the inclusion in paraffin can vary substantially and may hamper epigenetic studies in these samples. FA over-fixation is a common event which, similarly to what happens in canonical immunohistochemistry approaches, may interfere with antigen detection by the antibody and may decrease the amount of chromatin that can be isolated from FFPE sections.

We have experienced that the conduction of ChIP assays using low amounts of isolated input chromatin inevitably leads to poor DNA yields, particularly when low abundant epigenetic marks are investigated, and may negatively impact the subsequent library preparation resulting in higher rates of failure of epigenomic studies. In addition, the availability of low quantities of soluble chromatin limits the number of histone marks that can be studied in the same sample, requiring more starting FFPE material that is not always available in consequence of obvious ethical reasons.

The EPAT-ChIP protocol described here is characterized by the introduction of a controlled heat-mediated limited reversal of crosslinking (LRC) able to reduce tissue complexity, previously introduced by FA fixation, and thus to increase chromatin isolation efficiency from FFPE samples. Subsequent to the introduction of LRC, the original PAT-ChIP protocol was modified to avoid chromatin over-fragmentation by the removal of MNase chromatin digestion and the adjustment of sonication amplitude, with a consequent simplification of the entire protocol.

Attempts of changing the original PAT-ChIP protocol (Std) were conducted using normal human colon specimens as a proof-of-concept due to the availability of high quantities of this human material as scrap from colorectal surgeries, with also reduced ethical implications. The high availability of these tissues allowed us to fix specimens at different times (from 24 to 72 h), producing samples representative of what is normally found in FFPE archives.

We demonstrated that the duration of FA fixation progressively reduces the amount of chromatin that can be isolated from FFPE normal colon sections. The introduction of LRC is not only able to overcome the effect of long times of fixation on chromatin extraction efficiency but also to increase the amount of chromatin extractable at all the times of FA fixation tested and, most importantly, did not interfere with antigen recognition.

Once established that chromatin extracted with the introduction of LRC was compatible with the following immunoselection, using the low abundant H3K4me3 as a “stress-test” histone mark, we moved to another model of study to validate the new procedure. We chose an invasive breast carcinoma (IBC) as representative of real archival FFPE samples, and compared PAT-ChIP to the new EPAT-ChIP procedure.

We found that, also in this tissue, the efficiency of chromatin extraction is increased using EPAT-ChIP and, after H3K4me3 immunoselection, we obtained overlapping results at both locus-specific and genome-wide levels between the two procedures. In particular, we demonstrated (i) similar enrichments of expressed genes compared to inactive genes, (ii) comparable genomic distributions of the enriched regions, (iii) an almost complete overlap of peak-containing promoters identified by both PAT- and EPAT-ChIP, and (iv) sharper localization of peaks in TSS regions applying EPAT-ChIP. Regarding this last observation, it is reasonable to hypothesize that the reduction of tissue complexity, induced by LRC, could revert the negative impact of extensive fixation on the resolution of ChIP-Seq signals. To further support our results, we applied the same analytical pipeline that we used for our data sets to a H3K4me3 data set from HMEC, publicly available from UCSC Genome Browser. Surprisingly, despite the different experimental conditions used, the data sets showed a consistent distribution of peaks with respect to genomic features.

We then applied EPAT-ChIP to the epigenomic study of two additional HPTMs (H3K27me3 and H3K27ac) in the archival IBC FFPE sample, to extend the evidence of its applicability and robustness and to further compare the technique with the Std PAT-ChIP procedure. Again, we could observe the expected enrichments of gene promoters by qPCR using both H3K27me3 and H3K27ac antibodies. While the H3K27me3 ChIP-Seq profiles obtained using the two techniques were comparable, probably in consequence of the wide distribution of this HPTM that renders its immunoselection less critical with respect to other HPTMs, we observed a significant gain in the quality of H3K27ac profile when applying the EPAT-ChIP protocol compared to the Std technique.

To investigate a possible role of LRC in increasing antigen recognition by the antibody, we simulated a loss of binding caused by FA over-fixation using an in vitro setup and monitored the recovery of nuclear signals of H3K4me3, H3K27Ac, and H3K27me3 after the application of LRC (Additional file 5).

Other authors proposed alternative ways to overcome the hurdle of chromatin extraction from archival FFPE samples [35]. Considering the extreme variability in FFPE tissue processing, the rationale of our study was to improve the PAT-ChIP technology, in terms of chromatin isolation efficiency, to be applied in the most representative range of FA fixation (24–72 h) without the necessity of expensive instruments for chromatin extraction by sonication.

The EPAT-ChIP procedure has the potential to facilitate the application of epigenomic studies in clinical FFPE archival samples. This technique will allow genome-wide chromatin studies in pathology tissues, enabling its wide spread use and thus contributing to extend the current understanding of cancer epigenomes, the identification of novel tumor subtypes, and the development of new clinical biomarkers.

## Additional files

Additional file 1: **Figure S1.** H3K4me3 distribution at the promoter of inactive genes in the archival IBC sample. Snapshots of ChIP-Seq data from UCSC Genome Browser showing the absence of H3K4me3 enrichments in both standard PAT-ChIP (Std) and EPAT-ChIP (LRC) samples at promoters of the inactive genes HAPLN1 (**a**) and COL2A1 (**b**). CpG islands are reported as green bars and Ref-Seq genes are indicated in blue. (PDF 87 kb)

Additional file 2: **Figure S2.** Analysis of H3K4me3 ChIP-Seq data from ENCODE project. H3K4me3 data from human mammary epidermal cells (HMEC) was taken from UCSC Genome Browser and analyzed following the same pipeline used for standard PAT-ChIP and EPAT-ChIP (LRC) data sets. Pie charts depicting the distribution of peaks across genomic features with relative percentage values shown on the right (**a**). Heatmaps illustrating H3K4me3 peak densities from − 10 Kb to + 10 Kb relative to the TSS (**b**). (PDF 250 kb)

Additional file 3: **Figure S3.** DNA recovery after immunoselection with antiH3K27ac and anti-H3K27me3 antibodies. After immunoselection chromatin was de-crosslinked and the DNA purified and fluorimetrically quantified (**a**). Input fractions were also purified and quantified, and the percentage of enrichment by the antibody compared to the input was calculated (**b**). (PDF 43 kb)

Additional file 4: **Figure S4.** Analysis of H3K27me3 and H3K27ac ChIP-Seq data from ENCODE project. H3K27me3 (**a**) and H3K27ac (**b**) data set from human mammary epidermal cells (HMEC) was taken from UCSC Genome Browser and analyzed following the same pipeline used for Std PAT-ChIP and EPAT-ChIP (LRC) data sets. Pie charts depicting the distribution of peaks across genomic features with relative percentage values shown on the right. Promoters are defined as − 3 Kb to + 3 Kb relative to the TSS while Downstream as − 3 Kb relative to the end of 3’ UTR region. (PDF 45 kb)

Additional file 5: **Figure S5.** Effect of LRC on antigen recovery from highly-fixed cells. HeLa cells were subjected to formaldehyde fixation at standard conditions (1% FA, 10 min at + 37 °C—normal fixation) or to prolonged fixation (4% FA, for 4 h at + 37 °C—high fixation). Over-fixed cells were treated with LRC or left untreated. Cells were stained by immunofluorescence with anti-H3K4me3, anti-H3K27ac or anti-H3K27me3 antibody (green) following the same procedure described for the PAT-ChIP assay (buffers, timing and temperature of incubations). DAPI staining of nuclei (blue) is also shown. (PDF 1999 kb)

## Abbreviations

ChIP: Chromatin immunoprecipitation
EPAT-ChIP: Enhanced PAT-ChIP
FFPE: Formalin-fixed paraffin-embedded
HMEC: Human mammary epithelial cells
HPTMs: Histone post-translational modifications
IBC: Invasive breast carcinoma
IHC: Immunohistochemistry
LCM: Laser capture microdissection
LRC: Limited reversal of crosslinking
MNase: Micrococcal nuclease
NGS: Next-generation sequencing
PAT-ChIP: Pathology tissue-chromatin immunoprecipitation
qPCR: Quantitative PCR
Std: Standard
