# Supplementary material for: Epigenomic profiling of archived FFPE tissues by enhanced PAT-ChIP (EPAT-ChIP) technology
Source: Clin Epigenetics. 2018 Nov 16;10:143. doi: 10.1186/s13148-018-0576-y (PMC6240272; doi:10.1186/s13148-018-0576-y)
Supplement: Supplementary file 1 — Figure S1. H3K4me3 distribution at the promoter of inactive genes in the archival IBC sample. Snapshots of ChIP-Seq data from UCSC Genome Browser showing the absence of H3K4me3 enrichments in both standard PAT-ChIP (Std) and EPAT-ChIP (LRC) samples at promoters of the inactive genes HAPLN1 (a) and COL2A1 (b). CpG islands are reported as green bars and Ref-Seq genes are indicated in blue. (PDF 87 kb) [file 13148_2018_576_MOESM1_ESM.pdf]

Figure S1

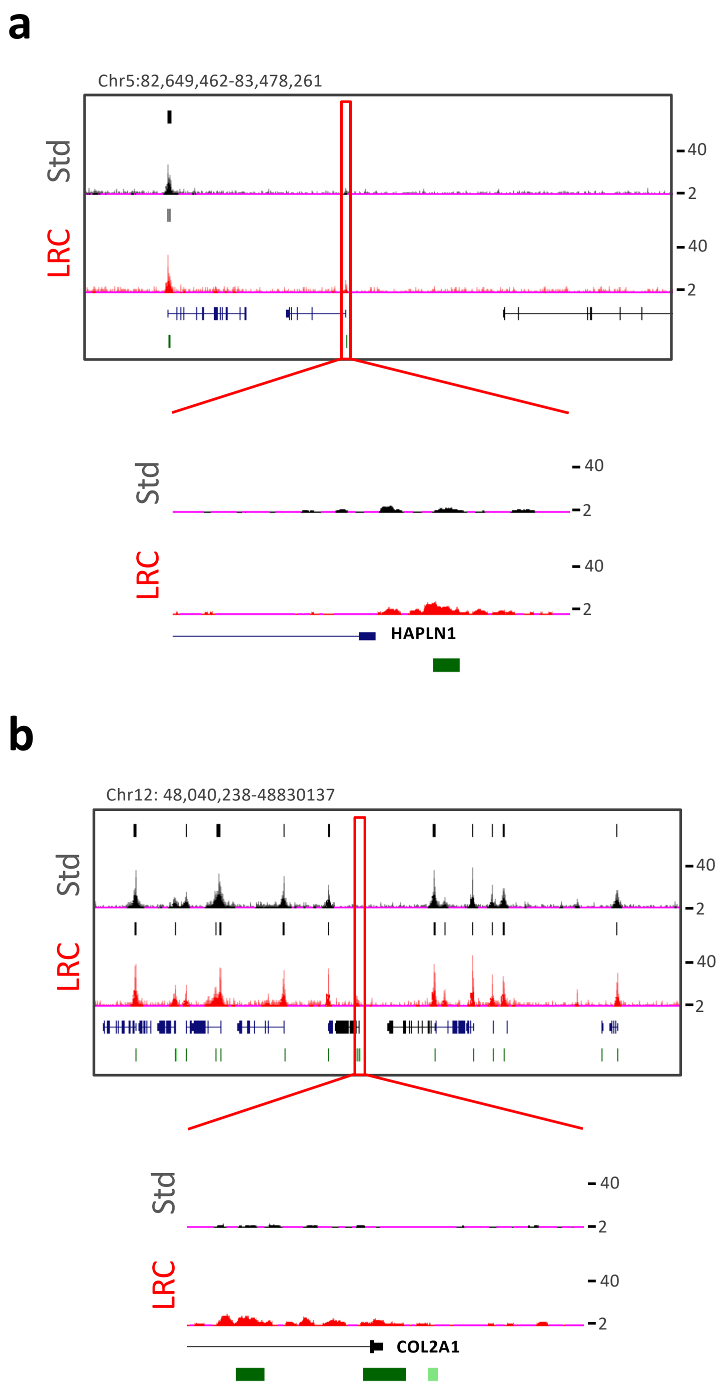

Figure S1. H3K4me3 distribution at the promoter of inactive genes in the archival IBC sample. Snapshots of ChIP-Seq data from UCSC Genome Browser showing the absence of H3K4me3 enrichments in both standard PAT-ChIP (Std) and EPAT-ChIP (LRC) samples at promoters of the inactive genes HAPLN1 (**a**) and COL2A1 (**b**). CpG islands are reported as green bars and Ref-Seq genes are indicated in blue.
