# Supplementary material for: Epigenomic profiling of archived FFPE tissues by enhanced PAT-ChIP (EPAT-ChIP) technology
Source: Clin Epigenetics. 2018 Nov 16;10:143. doi: 10.1186/s13148-018-0576-y (PMC6240272; doi:10.1186/s13148-018-0576-y)
Supplement: Supplementary file 2 — Figure S2. Analysis of H3K4me3 ChIP-Seq data from ENCODE project. H3K4me3 data from human mammary epidermal cells (HMEC) was taken from UCSC Genome Browser and analyzed following the same pipeline used for standard PAT-ChIP and EPAT-ChIP (LRC) data sets. Pie charts depicting the distribution of peaks across genomic features with relative percentage values shown on the right (a). Heatmaps illustrating H3K4me3 peak densities from − 10 Kb to + 10 Kb relative to the TSS (b). (PDF 250 kb) [file 13148_2018_576_MOESM2_ESM.pdf]

Figure S2

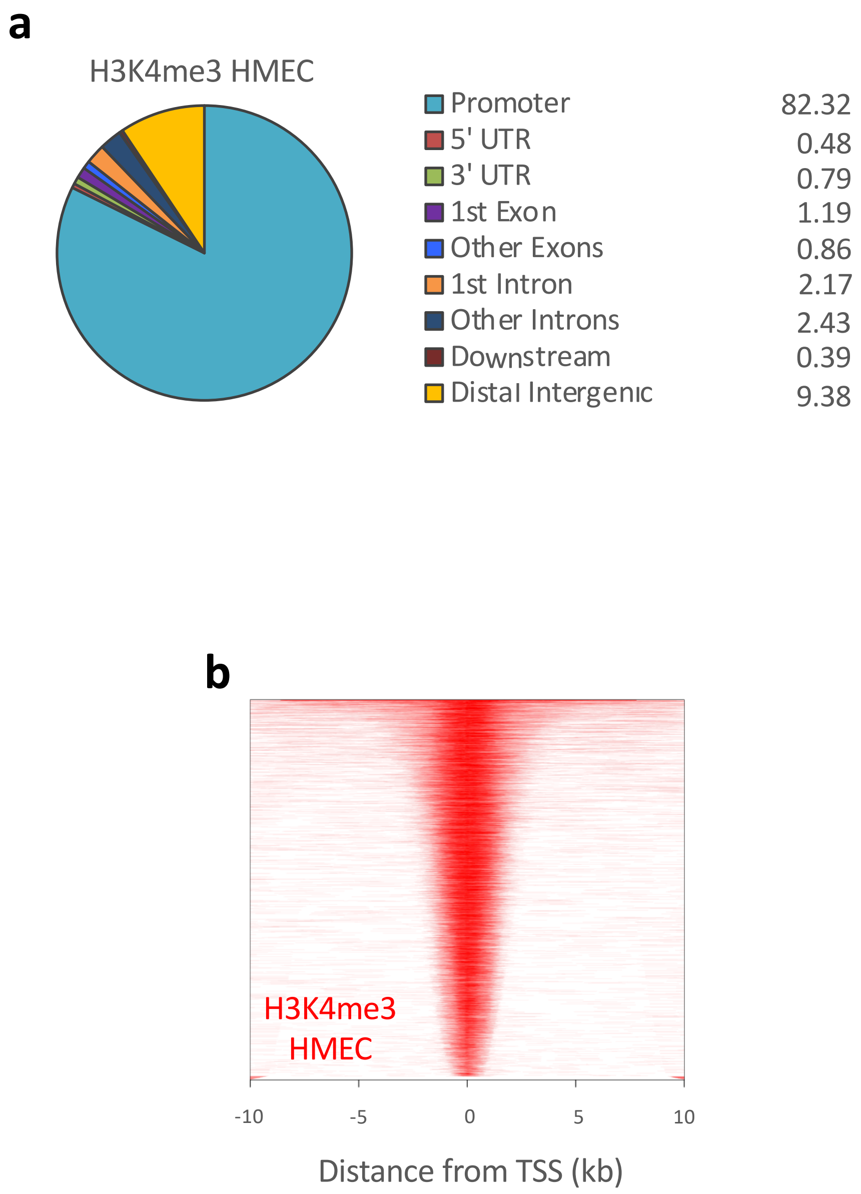

Figure S2. Analysis of H3K4me3 ChIP-Seq data from ENCODE project. H3K4me3 data from human mammary epidermal cells (HMEC) was taken from UCSC Genome Browser and analysed following the same pipeline used for standard PAT-ChIP and EPAT-ChIP (LRC) data sets. Pie charts depicting the distribution of peaks across genomic features with relative percentage values shown on the right (**a**). Heatmaps illustrating H3K4me3 peak densities from -10 Kb to +10 Kb relative to the TSS (**b**).
