# Supplementary material for: Epigenomic profiling of archived FFPE tissues by enhanced PAT-ChIP (EPAT-ChIP) technology
Source: Clin Epigenetics. 2018 Nov 16;10:143. doi: 10.1186/s13148-018-0576-y (PMC6240272; doi:10.1186/s13148-018-0576-y)
Supplement: Supplementary file 3 — Figure S3. DNA recovery after immunoselection with antiH3K27ac and anti-H3K27me3 antibodies. After immunoselection chromatin was de-crosslinked and the DNA purified and fluorimetrically quantified (a). Input fractions were also purified and quantified, and the percentage of enrichment by the antibody compared to the input was calculated (b). (PDF 43 kb) [file 13148_2018_576_MOESM3_ESM.pdf]

Figure S3

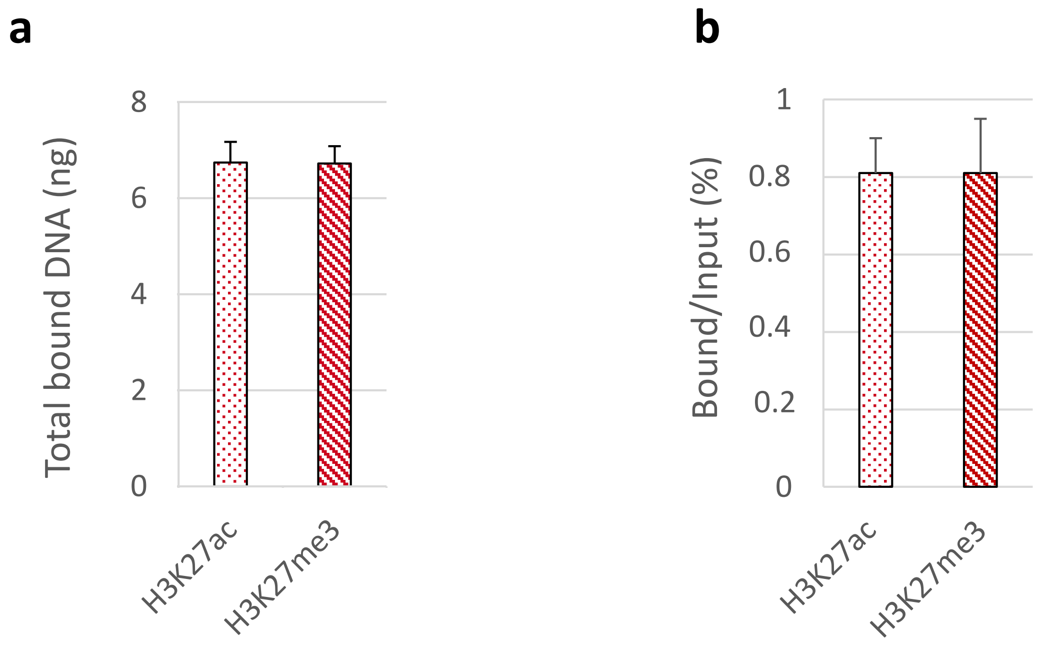

Figure S3. DNA recovery after immunoselection with antiH3K27ac and anti-H3K27me3 antibodies. After immunoselection chromatin was de-crosslinked and the DNA purified and fluorimetrically quantified (**a**). Input fractions were also purified and quantified, and the percentage of enrichment by the antibody compared to the input was calculated (**b**).
