# Supplementary material for: Epigenomic profiling of archived FFPE tissues by enhanced PAT-ChIP (EPAT-ChIP) technology
Source: Clin Epigenetics. 2018 Nov 16;10:143. doi: 10.1186/s13148-018-0576-y (PMC6240272; doi:10.1186/s13148-018-0576-y)
Supplement: Supplementary file 4 — Figure S4. Analysis of H3K27me3 and H3K27ac ChIP-Seq data from ENCODE project. H3K27me3 (a) and H3K27ac (b) data set from human mammary epidermal cells (HMEC) was taken from UCSC Genome Browser and analyzed following the same pipeline used for Std PAT-ChIP and EPAT-ChIP (LRC) data sets. Pie charts depicting the distribution of peaks across genomic features with relative percentage values shown on the right. Promoters are defined as − 3 Kb to + 3 Kb relative to the TSS while Downstream as − 3 Kb relative to the end of 3’ UTR region. (PDF 45 kb) [file 13148_2018_576_MOESM4_ESM.pdf]

Figure S4

a

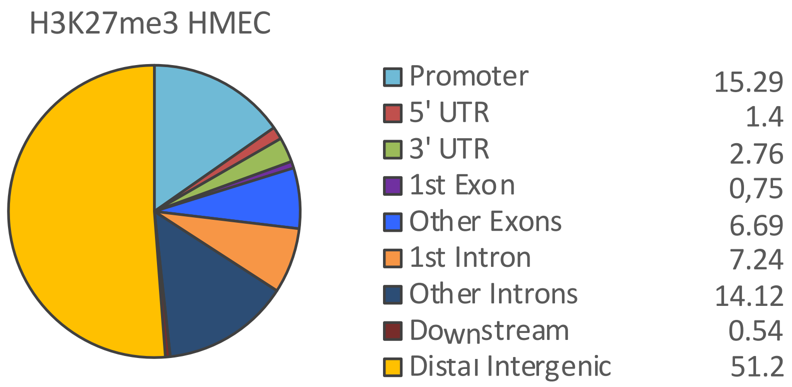

b

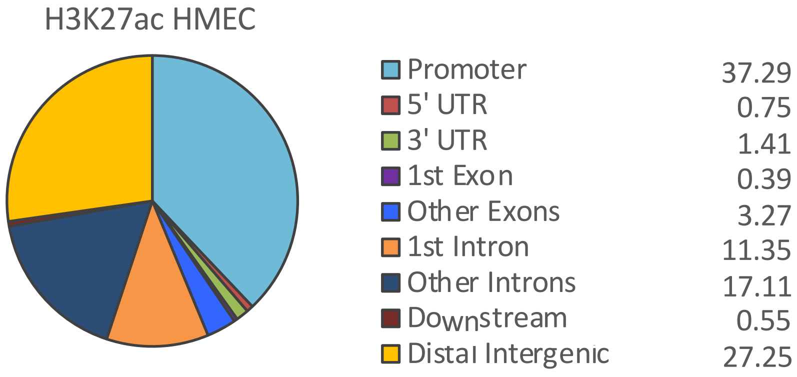

Figure S4. Analysis of H3K27me3 and H3K27ac ChIP-Seq data from ENCODE project. H3K27me3 (**a**) and H3K27ac (**b**) data set from human mammary epidermal cells (HMEC) was taken from UCSC Genome Browser and analyzed following the same pipeline used for Std PAT-ChIP and EPAT-ChIP (LRC) data sets. Pie charts depicting the distribution of peaks across genomic features with relative percentage values shown on the right. Promoters are defined as -3 Kb to +3 Kb relative to the TSS while Downstream as -3 Kb relative to the end of 3’ UTR region.
