# Supplementary material for: Epigenomic profiling of archived FFPE tissues by enhanced PAT-ChIP (EPAT-ChIP) technology
Source: Clin Epigenetics. 2018 Nov 16;10:143. doi: 10.1186/s13148-018-0576-y (PMC6240272; doi:10.1186/s13148-018-0576-y)
Supplement: Supplementary file 5 — Figure S5. Effect of LRC on antigen recovery from highly-fixed cells. HeLa cells were subjected to formaldehyde fixation at standard conditions (1% FA, 10 min at + 37 °C—normal fixation) or to prolonged fixation (4% FA, for 4 h at + 37 °C—high fixation). Over-fixed cells were treated with LRC or left untreated. Cells were stained by immunofluorescence with anti-H3K4me3, anti-H3K27ac or anti-H3K27me3 antibody (green) following the same procedure described for the PAT-ChIP assay (buffers, timing and temperature of incubations). DAPI staining of nuclei (blue) is also shown. (PDF 1999 kb) [file 13148_2018_576_MOESM5_ESM.pdf]

Figure S5

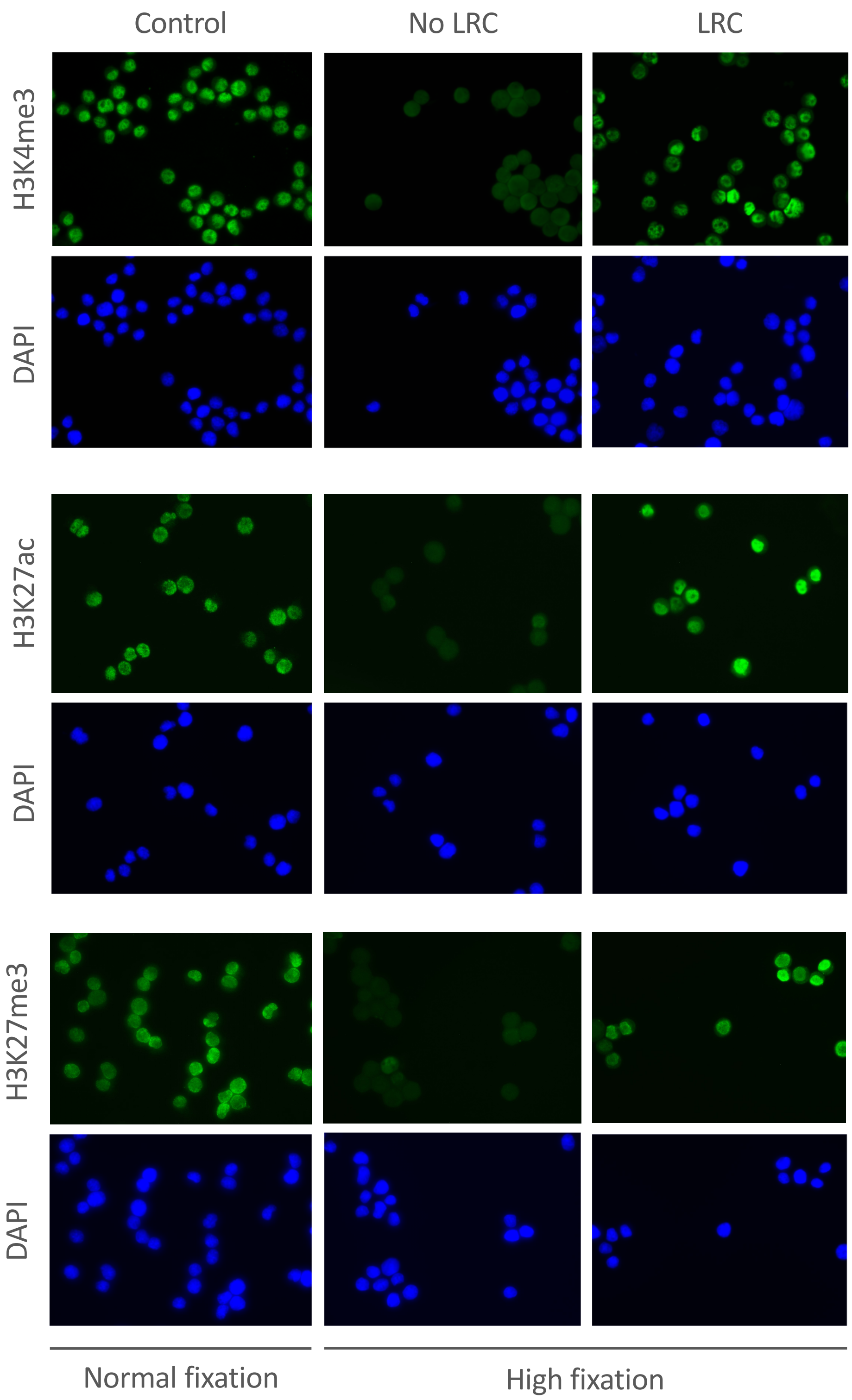

Figure S5. Effect of LRC on antigen recovery from highly-fixed cells. HeLa cells were subjected to formaldehyde fixation at standard conditions (1% FA, 10 min at +37 °C – normal fixation) or to prolonged fixation (4% FA, for 4 h at +37 °C – high fixation) to mimic over-fixation found in FFPE archival tissues. Over-fixed cells were treated with LRC or left untreated. Cells were stained by immunofluorescence with anti-H3K4me3, anti-H3K27ac or anti-H3K27me3 antibody (green) following the same procedure described for the PAT-ChIP assay (buffers, timing and temperature of incubations). DAPI staining of nuclei (blue) is also shown.
